# There is No Scope for Tobacco Funded Research in our Society

**DOI:** 10.1186/1617-9625-2-4-163

**Published:** 2004-12-15

**Authors:** Anthony J Hedley

**Affiliations:** 1 Department of Community Medicine, University of Hong Kong, Hong Kong, PR China

## 

The following position statement has been endorsed by the Executive Committee of the International Society for the Prevention of Tobacco Induced Diseases.

The *International Society for the Prevention of Tobacco Induced Diseases *is a Multidisciplinary group of tobacco control advocates comprising laboratory scientists, clinicians, health care researchers and public health workers. Their common aim is the prevention of recruitment of any individual to nicotine addiction and tobacco dependency, the promotion of smoking cessation and elucidation of the mechanisms and outcomes of injury caused by smoking.

A major objective of global tobacco control is to restrict the ability of transnational tobacco companies to promote their products, glamorize smoking in the eyes of young people, propagate misleading information about the harm of active and passive smoking and delay or dilute legislation.

In the third Annual Scientific Meeting of the Society in Louisville Kentucky, U.S., in October 2004, two of the posters gave acknowledgement to funding by the Philip Morris External Research Programme (PMERP). Although these are the only two such cases in the Society's three-year history, the Executive Committee regrets that this happened and wishes to reaffirm that there is no scope whatsoever for any tobacco sponsored or influenced activities within the Society. Although the Society's rules and intentions may not have been sufficiently clear, the Executive Committee has now resolved to never again accept tobacco funded research for presentation or publication.

While authors of tobacco funded research may deny any influence of their paymasters on their outputs, this is not the point. The tobacco industry is currently seeking to reposition itself, acquire new legitimacy, ingratiate itself with lawmakers and desperately avoid the impacts of both legislation and litigation which will damage their sales and budgets. The industry's defence lawyers state in the current trial in Washington, D.C., (under the Racketeering Influenced and Corrupt Organization Act-RICO), that the tobacco industry should be judged on its present behaviour, not its past history. The question is how much mendacity and anti-social behaviour can be swept under even large carpets?

As reported in the November issue of *Academic Medicine *[1], journal of the American Medical Colleges, the industry has recently "exploited institutional fears of losing research funding as part of its strategy to avoid tobacco stock divestment by US medical schools." As stated by Ruth Malone RN, PhD, senior author of the report and associate professor of nursing in University of California, San Francisco, School of Nursing, "Funding research is a way the industry tries to buy legitimacy. There are contradictions in selling off tobacco stocks while continuing to take money derived from tobacco profits." [1]

There are contradictions too for ISPTID. If we allow the presentation of tobacco funded research at our meetings we would have to pretend that the entire history of the corruption of science by big tobacco did not matter any longer.

In 1988 Philip Morris, Lorillard and RJ Reynolds formed the "Centre for Indoor Air Research" (CIAR) to support research on "indoor air quality." The aim was to distance the new organization of CIAR from the US Tobacco Institute and encourage scientists outside the industry to participate. The CIAR was wholly funded and controlled by the industry. Professor Richard Daynard of Northeastern University School of Law, Boston, Massachusetts, U.S., is quoted as saying, "Their true purpose was to generate disformation." [2]

As part of the 1998 US Attorneys General Master Settlement (Minnesota) with the industry, it was agreed that the US Tobacco Institute and the CIAR would be disbanded. Following this Philip Morris established the PMERP, in place of CIAR, in the same offices of the same town in Maryland, under the same director, Dr. Max Eisenberg. [3]

The aims of PMERP are apparently in large measure to counter public health advocacy for tighter tobacco control by being seen to be doing good. History suggests that ISPTID should not allow itself to be ensnared in this process.

In the late 1970s and 1980s the concealment and obfuscation of the harmful effects of both mainstream and sidestream (second-hand) smoke became a major project within the transnational tobacco companies. As described recently in *The Lancet *of November 11, 2004, Pascal Diethelm, Jean-Charles Rielle and Martin Mckee [4] used previously secret industry documents to dissect the covert operations of Philip Morris (PM). They describe how PM sought to inform themselves of the hazards of their products but deny or misrepresent their laboratory findings to others. PM even carefully hid the research operations from most of their staff. One former employee is quoted as stating in 1996 "I subsequently found out (by asking around) that hardly anyone [at Philip Morris] knew anything about INBIFO". INBIFO was the Institute für Industrielle und Biolgische Forschung GMbH which Philip Morris bought as part of its offshore research operation. A PM executive said its purchase would create "a locale where we might do some of the things which we are reluctant to do in this country" (i.e., the U.S.). Another senior PM executive said that research "on a contractual basis in Europe... presents an opportunity that is relatively lacking in risk and unattractive repercussions in this country."

Some of the unpublished research findings in INBIFO reports are staggering in terms of their clarity and implications. For example, as described by Diethelm and colleagues [4], the rat experiments involving exposures to either mainstream or sidestream smoke clearly delineate the biological toxicity of the different chemical profiles of these agents:

"All rats showed general signs of exhaustion after the end of the daily exposure. In contrast to the rats of the mainstream group, which recovered by next morning, the rats of the sidestream groups continued to show shaggy fur and some pronounced respiratory symptoms characterized by whistling and rattling sounds."

The report also concluded that:

• The mainstream total particulate matter (TPM) would have to be increased three fold to produce similar reactions to those seen with sidestream exposure.

• Sidestream smoke ("puffed or non-puffed") caused more severe atrophic and necrotic lesions of the olfactory epithelium and frequent squamous cell metaplasia in the ciliated epithelium of the nasal cavity.

• In terms of equal TPM concentration, sidestream smoke showed higher toxicity in terms of body weight development, food consumption, rectal temperature and respiratory frequency, than mainstream smoke.

Diethelm and colleagues state that these findings were discussed in a letter to Dr. Thomas Osdene, chief scientist at Philip Morris, by Professor Ragnar Rylander, formerly chair of Environmental Medicine at Gothenberg University, who also held an adjunct professorial position at Geneva University. Rylander was a longstanding consultant to Philip Morris receiving, from the 1980s, $150,000 a year in fees and other compensation. [2] Rylander stated to Osdene, "The histology demonstrates more advanced lesions in the nasal epithelium and hyper- and metaplasia in areas which are not affected by mainstream smoke. The extent of cornification observed in these animals has never been seen before." If the industry had published these findings 22 years ago, public health measures to protect children, workers and the general public from secondhand smoke might be well advanced and institutionalized world-wide. Instead the industry has spent hundreds of millions of dollars to block and disable even the most basic protective measures against passive smoking. The consequences can be counted in illness episodes, hospital admissions, premature deaths and a massive cost to communities.

As stated in the conflict of interest statement in the *Lancet *article, Professor Rylander took legal action against two of the authors of the *Lancet *paper after they publicly exposed his covert activities for the tobacco industry. After three court cases including two appeals, judgment was given in favour of the defendants, Diethelm and Rielle. The court considered that Rylander had perpetrated "unprecedented scientific fraud." In a recent review by a special ethics committee in Geneva University, chaired by Professor Alex Mauron, its conclusion was:

"Considering his close association with the tobacco industry and the duplicity of his attitude throughout his professional career, it appears that Ragnar Rylander was not able to preserve his intellectual independence in the face of specific commercial interests. Documentary evidence leads one to believe that the attitude he adopted in his professional life consisted in unilaterally defending the interests of the tobacco industry in the conflict pitting the latter against scientists convinced of the harmful effects of passive smoking. He helped to elaborate the industry's strategy and in his agreements with the industry accepted a secrecy clause that led him to suppress information known to him regarding the toxic effects of smoking. The Commission considers it legitimate to doubt the validity of the body of Ragnar Rylander's work directly or indirectly concerned with tobacco smoke."

Professor Rylander of course rejects these findings. The Geneva court judgement and Geneva University enquiry report are now on the ISPTID website so that members may form their own views.

While we are not suggesting that any colleagues who have taken PMERP funds are intellectually dishonest, the history of tobacco funding of research shows such a large systematic bias in some of the outputs that we cannot, at this stage, have any confidence in tobacco funded activities.

Questions remain as to the extent to which tobacco control advocates should in the future participate in engagements with any arm of the industry or those who are funded by them. A group of about 75 people from tobacco sectors, together with eight from the industry met recently in New Orleans to address the questions:

1. What are the potential risks and benefits of tobacco industry sponsorship of scientific research?

2. Are there procedures or mechanisms that could help protect against the types of research abuse that have occurred in the past and that some believe are continuing to occur?

A report by Mitchell Zeller [5] on Globalink States:

"A number of issues and ideas were discussed by the group. The need for far greater transparency by the tobacco industry in the area of research funding was discussed. The Workshop also explored the theoretical possibility of creating an independent institution to distribute funds for research, including funds received from the tobacco industry. This independent institution would, of course, need to be free of influence from the tobacco industry in its operation in order to be credible and effective. However, no consensus was reached on the feasibility or desirability of this idea.

The Planning Committee believes that the Workshop objectives were met. Different viewpoints on this controversial topic were aired in a civil and respectful manner. Participants hopefully came away better informed as a result of their attendance at the Workshop. We firmly believe it is worthwhile to promote open discussion of the ethical, legal and policy issues of tobacco industry funding of tobacco research. Opinions across the entire spectrum are a welcome part of that dialogue and we are quite satisfied that our forum was a success in meeting that goal."

In the meantime and in the absence of an alternative formal approach, ISPTID will take all possible steps to distance itself from tobacco sponsorship in any form. It is a clearly stated rule of the Society that all conflicts of interest must be declared and tobacco funded projects will not be accepted for presentation or publication in any form.

The future of the prevention of tobacco induced disease critically depends on the prevention of the industry usurping any new and bogus role in tobacco control. Whether any alternative and safe model based on tobacco funding can be developed remains to be seen.

## Competing interests

The authors declare that they have no competing interests.

## References

[B1] Wander N, Malone RE (2004). Selling off or selling out?. Medical schools and ethical leadership in tobacco stock divestment Academic Medicine.

[B2] Thomas H, Gagliardi J Tobacco smoking guns. Hong Kong: South China Morning Post, Monday January 18, 1999 Monday focus.

[B3] Max Eisenberg Philip Morris External Research Program, Research Management Group, 1099 Winterson Road, Suite 280, Linthicum Heights, Maryland, 21090-2216, USA.

[B4] Diethelm PA, Rielle J-C, McKee M The whole truth and nothing but the truth?. The research that Philip Morris did not want you to see Lancet published online November 11, 2004.

[B5] Zeller M (2004). Issues raised by the offer and acceptance of tobacco industry funds for research Globalink.

